# A commentary on the paper, ‘Development and validation of a novel automatable assay for cholesterol efflux capacity’

**DOI:** 10.1042/BSR20230124

**Published:** 2023-06-05

**Authors:** Aishwarya Sudam Bhale, Krishnan Venkataraman

**Affiliations:** Centre for Bio-Separation Technology, Vellore Institute of Technology, Vellore 632014, Tamil Nadu, India

**Keywords:** cardiovascular disease (CVD), Cholesterol efflux capacity (CEC), dysfunctional HDL, High-density lipoprotein (HDL), High-density lipoprotein cholesterol (HDL-C), magnetic beads

## Abstract

The determination of functionality or quality of high-density lipoproteins (HDL) is assuming a central stage in the prediction of cardiovascular diseases (CVD). To assess HDL quality, several attempts have been made to develop an automated, cost-effective cholesterol efflux capacity (CEC) system with few operational steps that might be used in clinical settings for large throughput testing. The work of Dr. Ohkawa and co-workers seems to address this issue and provide a solution for the same (Bioscience Reports (2023), 43 BSR20221519, https://doi.org/10.1042/BSR20221519). Earlier work from the author’s lab utilized a radioisotope and cell-free CEC assay known as the immobilized liposome-bound gel beads (ILGs) method. However, this assay required a centrifugation step to separate the cells and was not suitable for automation. To overcome these limitations, two very important changes were made: (i) magnetic beads were used instead of gel beads that allowed them to avoid the centrifugation process that would allow ease of setting up an autonomous analyzer; (ii) porous magnetic beads were coated with liposomes containing fluorescently tagged cholesterol instead radiolabeled cholesterol. These two changes can be considered not only significant but also novel as they were highly suitable for CEC testing. The authors reported the successful development of a simple immobilized liposome-based magnetic beads (ILMs) automated system to measure CEC, which provided both consistent performance and satisfactory correlation with the other methods. Thus, we feel the present study will open newer avenues for measuring the quality of HDL in addition to the quantity of HDL-cholesterol in clinical settings in a more robust way.

## Commentary

Despite gains in living standards and medical technology-driven improvements in cardiovascular diseases (CVD) outcomes, the high incidence of CVD remains a significant health problem [1]. Ischemic heart disease and stroke, both clinical manifestations of atherosclerosis, account for 84.9% of fatalities from CVD and are the leading worldwide killers [2]. Lipids build up in the arterial wall during atherogenesis, causing inflammatory reactions that speed up the development of atherosclerosis [3]. Increased levels of low-density lipoproteins (LDL) are linked to the development of atherosclerosis [4,5]. It is fiercely disputed whether decreased high-density lipoproteins (HDL)-cholesterol, which is often used as a marker for CVD risk, actually causes CVD [6]. According to recent research, HDL-cholesterol only correlated with CVD risk in people who had no prior history of the disease. In addition, a U-shaped connection between total mortality and HDL-cholesterol was discovered in another study involving more than a million American veterans [7].

**Figure 1:**
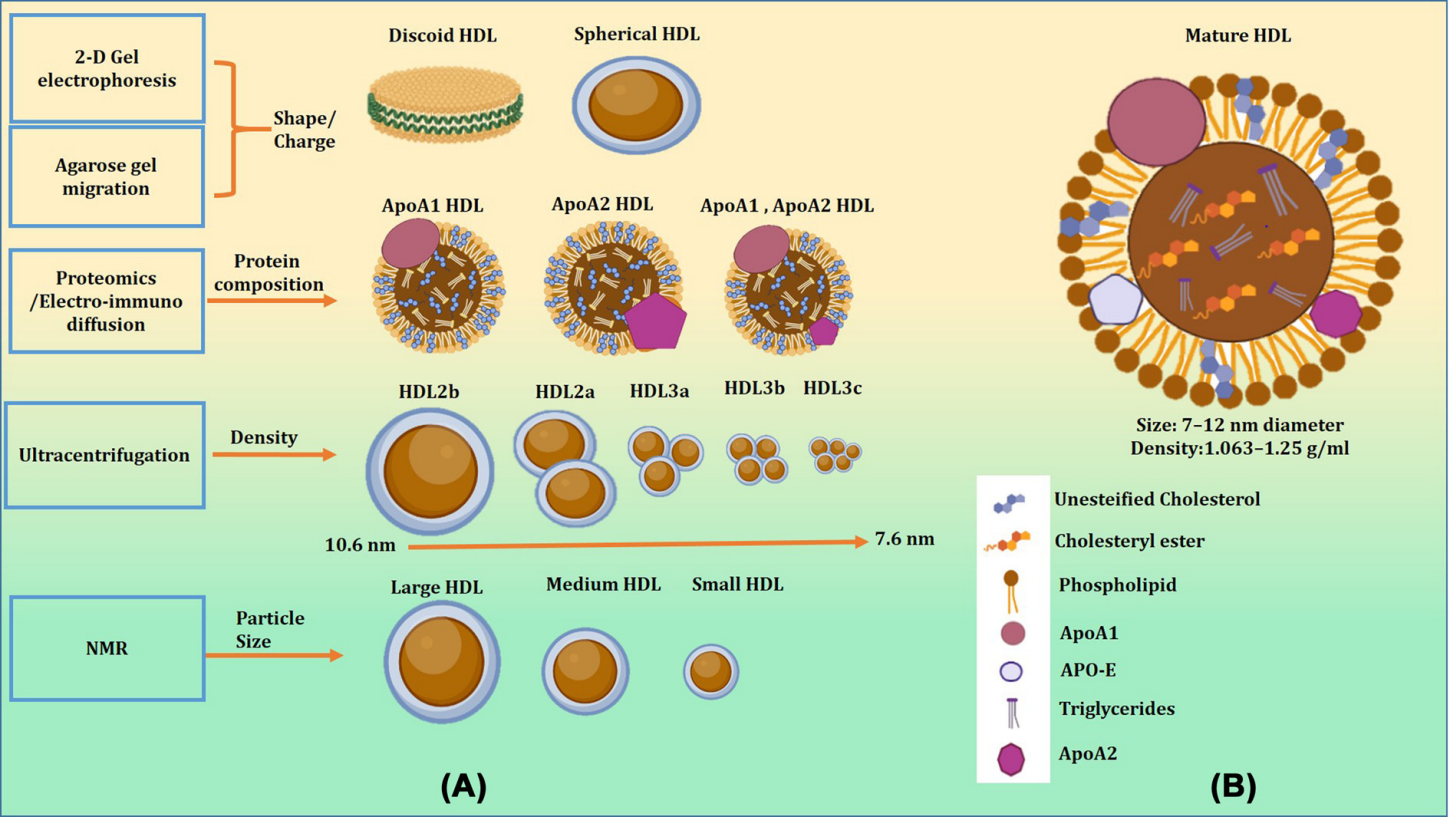
In terms of structure, content, and biological roles, HDL represents a class of lipoproteins that is incredibly diverse The density of HDL ranges from 1.063 to 1.25 g/ml. The heterogeneity of the HDL population is described using various techniques, highlighting the diversity of HDL subtypes.

**Figure 2:**
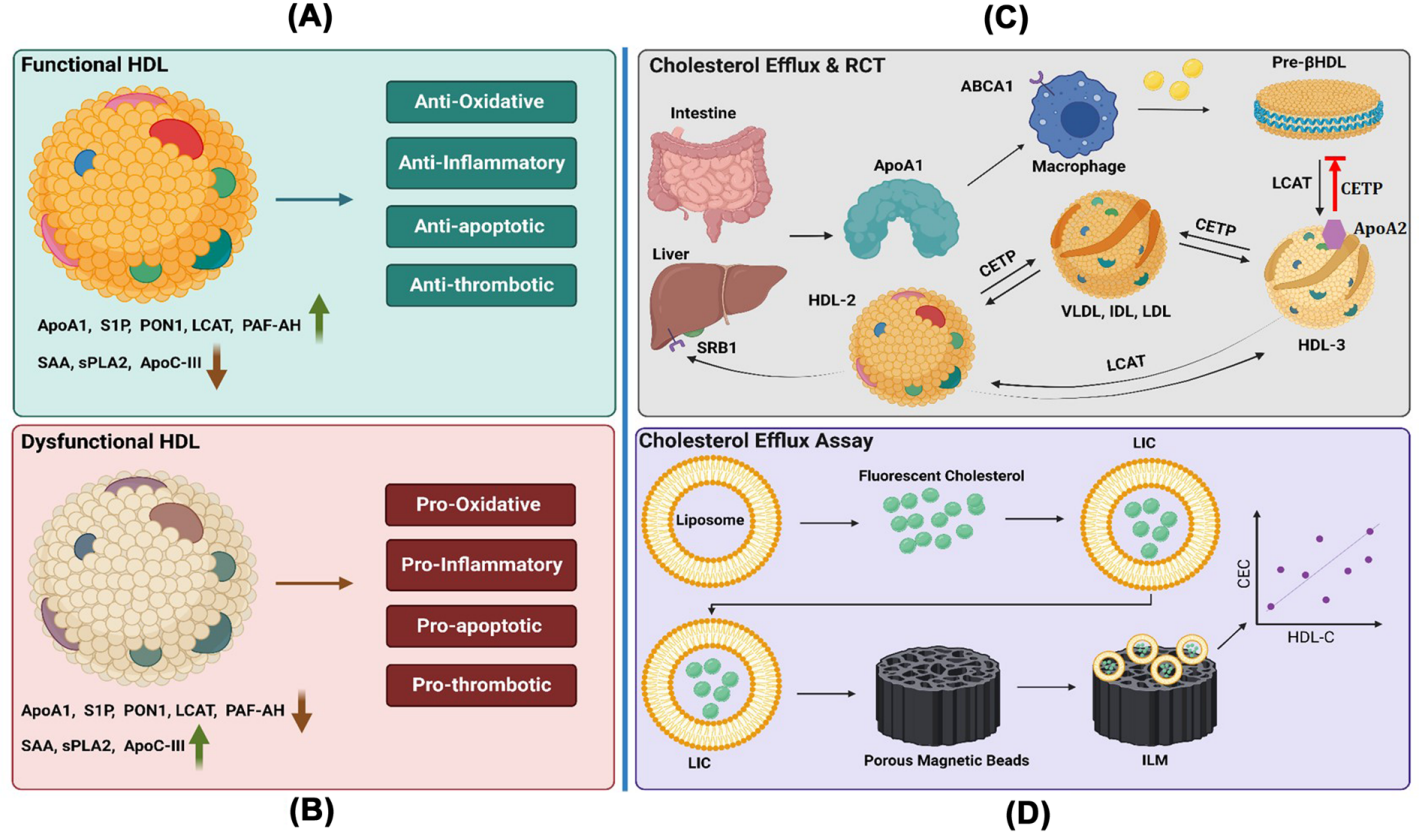
Illustration of Functional HDL, dysfunctional HDL, HDL biogenesis and cholesterol efflux assay (**A**) Functional HDL contains increased levels of ApoA1, sphingosine 1-phosphate (S1P), paraoxonase 1 (PON1), lecithin cholesterol acyl transferase (LCAT), and platelet-activating factor acetylhydrolase (PAF-AH). All these key proteins of HDL play important role in decreasing its dysfunctional cargo such as serum amyloid A (SAA), secretary phospholipase A2 (sPLA2), and Apolipoprotein C-III (ApoC-III). HDL with the above-mentioned cargo is generally considered functional HDL that maintains a healthy cardiovascular system. (**B**) In CVD, HDL that was formerly functional has become dysfunctional. Numerous proteins are altered by post-translational processes or mutations brought on by tissue damage and inflammation. Such dysfunctional HDL contains decreased ApoA1, S1P, PON1, LCAT, and PAF-AH. Such dysfunctional HDL also contains an increase in dysfunctional cargo such as SAA, sPLA2, and ApoC-III. (**C**) Cholesterol efflux and reverse cholesterol transport (RCT) is an important mechanism of HDL, which shows its ability to efflux cholesterol. The liver and intestines produce ApoA1, which becomes pre-βHDL after interacting with ATP-binding cassette receptor A1 (ABCA1). This interaction facilitates the efflux of cholesterol and phospholipids from ABCA1 to ApoA1 and the formation of pre-βHDL. Later ApoA1 of pre-βHDL activates LCAT resulting in the conversion of unesterified cholesterol to cholesteryl ester and producing spherical HDL3. Additionally, HDL3 goes through lipidation, producing HDL2 as a result. HDL2 can supply cholesterol for bile excretion by interacting directly with the liver’s SRB1 receptor. In addition, HDL2 and HDL3 can interact with cholesterol ester transfer protein (CETP), facilitating the transfer of triglycerides and cholesterol to other lipoproteins (such as very LDL (VLDL), LDL, and intermediate-density lipoprotein (IDL)). Also, some HDL particles contain both ApoA1 and ApoA2 (HDL3). Understanding the interaction between ApoA1 and ApoA2 proteins is necessary. ApoA2 blocks the dissociation of ApoA1 to produce pre-βHDL and the remodeling of HDL by the CETP. ApoA2 inhibits ApoA1’s ability to desorb from the HDL particle surface by interacting with it. (**D**) Liposomes containing fluorescently tagged cholesterol (LIC) were coated on porous magnetic beads in the Mutsuda et al.’s ILG approach. The magnetic beads with fluorescence successfully immobilized about the same number of liposomes as the ILG technique. According to the results, these ILM were appropriate for CEC testing. Understanding the connections between ApoA1 and ApoA2 proteins is necessary since some HDL particles include both ApoA1 and ApoA2 proteins. ApoA2 blocks the dissociation of ApoA1 to produce pre-β-HDL and the remodeling of HDL by the CETP. Through interactions with ApoA1, ApoA2 lessens the latter’s capacity to desorb from the HDL particle surface.

HDL represents a class of lipoproteins that is extraordinarily diverse in terms of structure, content, and biological functions [8]. Different methods are utilized to describe the HDL population, underscoring the variability of HDL subtypes [9] (Figure 1). HDL is the smallest lipoprotein with the highest density because of its high protein content [10]. Apolipoprotein A1 (ApoA1) makes up 70% of the protein mass in HDL, making it the main protein component [11]. The residual mass of HDL is completed by lipid transfer proteins like Lecithin cholesterol acyltransferase and, Cholesteryl ester transfer protein [12]. Furthermore, it contains 14 different types of Apolipoproteins and enzymes like paraoxonase 1 (PON1), and platelet-activating factor acetylhydrolase (PAF-AH), etc. [13] (Figure 2A). These HDL molecules are believed to inhibit LDL oxidation and prevent atherosclerosis [14]. In addition to proteins, HDL is enriched with lipid molecules like phospholipids, sphingolipids, neutral lipids, and minor lipids [15].

Reverse cholesterol transport (RCT) is one of the important functions of HDL that allows cholesterol efflux and transport back into the liver from peripheral cells [16]. It is a mechanism by which the proteins on HDL interact with numerous cell receptors and enzymes in a manner that favors the remodeling of HDL, one of the finest and most crucial mechanisms for safeguarding cardiovascular health [17] (Figure 2C).

Recent research suggests that increased HDL-cholesterol levels may not always be beneficial and that HDL can lose its cardioprotective properties due to malfunction [18]. In healthy individuals, HDL acts as an anti-inflammatory particle. However, HDL may actively encourage the inflammatory response in patients with chronic diseases [19]. Functional HDL contains significant amounts of anti-inflammatory proteins and enzymes [20]. Conversely, HDL can turn out to be a dysfunctional, proinflammatory particle that cannot facilitate cholesterol efflux or stop LDL oxidation when its antioxidant and anti-inflammatory properties are outweighed by pathological processes like inflammation [17]. These dysfunctional HDL particles exhibit decreased levels of anti-inflammatory factors and increased levels of proinflammatory markers [16] (Figure 2B). All these are signs of dysfunctional HDL, implying that the HDL quality may not be reflected by its cholesterol content. To know the HDL’s quality, we need to test HDL’s composition and functionality. Although there are reliable procedures for determining HDL’s heterogenity (Table 1), but here are no widely used assays exist in clinical settings to determine HDL quality [21]. Several research labs have developed *in vitro* tests for HDL function, but they lack standardization and are insufficiently confirmed.

**Table 1:** Techniques for HDL isolation: Methods and limitations

Sr. No.	HDL-cholesterol measurement parameters	Limitations of the technique
*1*	Colorimetric assay (Enzymatic)	Incomplete precipitation of Apo B
*2*	Ultracentrifugation	Expensive and technically complex
*3*	High-performance liquid chromatography (HPLC)	Expensive and technically complex
*4*	Non-denaturing polyacrylamide gradient gel electrophoresis	Standardization varies between laboratories
*5*	Two-dimensional gradient gel electrophoresis	Specialized laboratories are required
*6*	Nuclear magnetic resonance spectroscopy (NMR)	Specialized equipment is needed, and the methodology is complex
*7*	Raman instruments coupled with microscopy	Specialized equipment is needed, and the methodology is complex
*8*	CEC assays (dependent on differentiated cells and radioisotope-labeled cholesterol)	Complicated handling and safety

The authors of the research we are referencing, note that the traditional CEC measurement technique necessitates cultured cells and radioisotope-labeled cholesterol, both of which are impractical in clinical settings given large number of clinical samples to be analyzed. In their prior study, they created a CEC assay to circumvent this issue by using gel beads and apolipoprotein B-100-depleted serum (BDS) in place of cells and radioisotopes [22]. Unfortunately, their earlier procedure required the centrifugation step to spin down the gel beads and to separate cholesterol from supernatant that was fluorescently labeled [23]. The authors reported that, because gel beads quickly swirl up with light vibrations, extra care must be taken to prevent the interference from gel particle dust during the sample preparation step before centrifugation. As a result, they recommended that the assay was not appropriate for the installation of an automated analyzer. They created an improved method that eliminated the need for centrifugation steps during the assay to overcome the drawback produced by the previous method. They focused on magnetic beads since they are frequently employed to separate free and antibody-bound components in automatic immunoassay analyzers (Figure 2D).

In the initial methodology, they started by choosing special porous magnetic beads with pores that served the same purpose as gel beads in retaining liposomes. They were effective in attaching fluorescently labeled cholesterol to the magnetic beads. Fluorescence microscopy was used to examine treated magnetic beads. Green fluorescence was seen as spots on the beads, demonstrating that the BODIPY-labeled cholesterol-containing liposomes penetrated the beads and remained there. They also calculated the amount of liposomes that were immobilized on the magnetic beads. The fluorescence intensity of each supernatant was determined after collecting the supernatants before and after a number of freeze-thaw cycles. The amount of fluorescence that was found after three freeze-thaw cycles was 22.26 (AU); however, fluorescence increased after five, seven, and nine freeze-thaw cycles (25.44, 31.01, and 32.49 (AU), respectively). Additionally, the results showed that the liposomes formed inside the magnetic beads during freeze-thaw cycles rather than on the surface, which was confirmed by florescence microscopy.

In terms of the immobilization rate, the number of immobilized liposomes was higher in the ILM method than in their previous study (ILG). They reported that the ILM approach has a higher liposome immobilization efficiency than the ILG method. Before ILM is used in the laboratory diagnostic settings, its stability should also be examined. The authors, therefore, assessed the ILM’s stability and their findings showed that the ILM solution was stable for 45 days at 4 degrees [22].

They estimated the CEC of diluted HDL or BDS samples to investigate the fundamental accuracy of CEC measurement using ILM. Their findings demonstrated that CEC increased in both cases in a concentration-dependent manner. However, linearity was not demonstrated in extremely diluted samples for both HDL and BDS. According to the authors, this is because the amount of cholesterol that HDL was able to extract was insufficient for measuring CEC in the diluted samples. The authors also showed that the CEC values derived by the ILG and ILM approaches had a strong correlation. The CEC of the ILM approach was lower in the HDL samples compared with BDS. This may be due to the different efflux capacities of HDL and BDS. In contrast with HDL, BDS comprises a variety of serum proteins, including globulin. It is known that globulin can efflux cholesterol in the ILG technique. As a result, this difference might have happened because these serum proteins extracted cholesterol from the ILM in the case of BDS [24].

Later, the amount of CEC in the serum of 15 healthy subjects with varied HDL-cholesterol values were assessed by the ILM method. First, they discovered that low HDL cholesterol (<1.2 mg/dl) was below the detection limit when they tried to perform CEC measurements by the ILM method using 2% BDS. They also discovered that precise measurement of serum samples from healthy subjects with low HDL cholesterol concentration (<60 mg/dl) was not possible when it was done using 2% BDS. In order to assess blood HDL cholesterol concentrations up to 34 mg/dl, they chose to measure the CEC of healthy participants at a BDS concentration of 3.5%. which gave the consistent and reproducible results. As a result, the ILG technique and CEC values obtained using the ILM method (3.5% BDS) displayed a quite high correlation. Notably, some BDS samples from patients with similar HDL-cholesterol concentrations had significant variations in CEC values, suggesting that a given amount of HDL had a different antiatherosclerotic capacity.

Both ILM and ILG procedures do not use cellular sources as cholesterol donors, and there will be a change or fluctuation in different batches, which was the study’s limitation. Although the authors note that utilizing reference BDS to correct the CEC values obtained from individual experiments is an effective strategy, but still requires a manual precipitation step. Instead of just measuring HDL-cholesterol, many features of HDL structure and function, particularly in RCT, maybe more accurate predictors of HDL’s protective action. One of the remaining limitations of the ILM technique is that, it does not account for the CEC of lipid-free ApoA1. Whether the CEC values generated by the ILM accurately represent the efflux capacity through the cellular transporters (ABCA1, ATP-binding cassette receptor G1) *in vivo* is another unanswered subject. Therefore, it is crucial to examine these CEC events using the ILM approach before using them in future clinical studies on a diverse clinical samples.

## Conclusion and future direction

The present study by Mutsuda et al. showed the liposomes containing cholesterol that was fluorescently tagged and successfully immobilized on magnetic beads [22]. They developed a novel CEC assay, which does not require the centrifugal step. Furthermore, they concluded that this approach may be utilized to assess CEC in patient samples. It is highly required to confirm whether CEC calculated using the ILM approach can be assessed in different patient types and can identify different classes of HDL and its nature as per the cargo. Finally, the reliability of this technology in evaluating the CEC as a measure of the quality of HDL in large cohorts of samples across various populations and their outcome may help in adopting this technology in routine clinical settings.

## Abbreviations

ABCA1: ATP-binding cassette receptor A1
ApoA1: apolipoprotein A1
ApoC-III: apolipoprotein C-III
AU: fluoroscence unit
BDS: apolipoprotein B-100-depleted serum
CEC: cholesterol efflux capacity
CETP: cholesterol ester transfer protein
CVD: cardiovascular disease
HDL: high-density lipoprotein
ILG: immobilized liposome-bound gel bead
ILM: immobilized liposome-bound magnetic bead
LCAT: lecithin cholesterol acyl transferase
LDL: high-density lipoprotein
PAF-AH: platelet-activating factor acetylhydrolase
PON1: paraoxonase 1
RCT: reverse cholesterol transport
S1P: sphingosine 1-phosphate
SAA: serum amyloid A
sPLA2: secretary phospholipase A2
